# Left atrium volume and function changes during stress in patients with primary mitral regurgitation and preserved left ventricular ejection fraction

**DOI:** 10.1177/02676591241251441

**Published:** 2024-04-30

**Authors:** Rūta Dirsienė, Rugilė Martinaitytė, Eglė Tamulėnaitė, Aistė Montvilaitė, Dainius Karčiauskas, Eglė Ereminienė, Justina Jolanta Vaškelytė

**Affiliations:** 1 Faculty of Medicine, 230647 Lithuanian University of Health Sciences, Kaunas, Lithuania; 2 Institute of Cardiology, 230647 Lithuanian University of Health Sciences, Kaunas, Lithuania

**Keywords:** primary mitral regurgitation, left atrium, stress echocardiography, speckle-tracking echocardiography

## Abstract

**Introduction:**

Patients with primary mitral regurgitation (MR) usually remain asymptomatic for a long time due to compensatory mechanisms and an adequate treatment could be delayed. Stress echocardiography and speckle-tracking analysis could help to evaluate impaired left atrium (LA) function before the manifestation of clinically significant myocardial changes in asymptomatic patients with primary MR and preserved left ventricular (LV) ejection fraction (EF).

**Methods:**

This study prospectively enrolled 91 patients with preserved LV EF (≥60%) at rest, of which 60 patients had moderate-to-severe MR and 31 were healthy controls. Rest and stress (bicycle ergometry) echocardiography and speckle-tracking offline analysis were performed.

**Results:**

In MR group LA volume indices were higher at rest and during stress, while LA reservoir, conduit, and contractile fractions were decreased (*p* < .005). LA deformation parameters at rest were similar in both groups. During maximum stress LA conduit, contractile fractions and reservoir strain were lower (*p* < .05) in patients with MR. Indices of LA volume were related to SPAP at rest and during stress. Higher NT–proBNP concentrations was associated with higher LA volume indices, decreased contractile and reservoir functions during peak stress (*p* < .05). LA volume indices, LA EF, and filling index at rest could predict exercise-induced pulmonary hypertension (EIPH) (*p* < .05).

**Conclusions:**

In patients with primary MR and preserved LV EF, LA parameters are related to SPAP and NT-pro-BNP concentration. LA volume indices, LA EF and LA filling index are predictors of EIPH.

## Introduction

Mitral regurgitation (MR) is the second most prevalent valvular disorder requiring surgery worldwide.^1^ Echocardiography is crucial for diagnosing MR, evaluating its mechanism, assessing the severity and potential complications of a disease, including left ventricle (LV) and left atrium (LA) dilation, LV dysfunction, and pulmonary hypertension (PH).^2^

In the past decade, stress echocardiography has become more widely used for various indications, including asymptomatic primary MR.^3,4^ The main purpose of exercise echocardiography in these patients is to provoke symptoms, increase systolic pulmonary artery pressure (SPAP), detect LV dysfunction, and stratify the risk,^5^ given that the presence of symptoms is usually a delayed indication for surgery.^2,6^ To enhance survival rates, assessing the need for surgery before irreversible myocardial changes occur is crucial.^7^ According to current guidelines, elevated SPAP, dilated LA and development of atrial fibrillation have negative prognostic implications and indicate consideration for surgical demand.^2,6,8^

During the early phase of MR, the increased regurgitant volume is compensated by remodelling and enlarging the LA, which becomes the primary target for MR damage.^9^ Previous studies proved that LA size is not only directly related to increased LV filling pressure and acts as a marker of the severity of LV diastolic dysfunction,^10^ but also reflects the cumulative long-lasting effect of filling pressure while Doppler indices of diastolic function show filling pressure at exactly one point in time.^10^ Another sensitive and reproducible method to evaluate LA mechanics is speckle-tracking echocardiography (STE)^11^: LA strain imaging can detect impaired LA function even before the manifestation of LA structural changes.^9,12^

The aim of this study was to evaluate LA size and function during the exercise stress and to find out a correlation between LA parameters and exercise-induced PH (EIPH), NT-proBNP values in patients with moderate to severe primary MR and preserved LV EF.

## Methods

### Study population

We prospectively studied 91 patients with preserved LV EF (≥60). 60 (65.9 %) adult patients with primary moderate to severe MR (MR group) were enrolled in the study and 31 (34.07 %) age-matched healthy patients were recruited as controls. The MR group was further subdivided based on EIPH (SPAP >60 mmHg during peak stress). One-third of the MR sample (*n* = 20; 33.33 %) had EIPH (PH subgroup). The study protocol was approved by the Kaunas Regional Biomedical Research Ethics Committee (Project ID: BE 2-54, approval date: June 26, 2018).

Patients were excluded from the study with at least one of the criteria: (1) contraindications or inability to perform bicycle ergometry; (2) clinically significant ischemic heart disease; (3) active oncological process; (4) asthma, chronic obstructive pulmonary disease or other known lung pathology; (5) uncontrolled arterial hypertension; (6) significant LV hypertrophy (thickness of interventricular septum or posterior LV wall >13 mm); (7) previous cardiac surgery; (8) greater than mild aortic valve disease (stenosis or regurgitation), more than mild mitral stenosis.

### Transthoracic echocardiography

All study subjects underwent transthoracic two–dimensional echocardiography at rest and during exercise (bicycle ergometry). Echocardiography was performed by an experienced echocardiographer using a diagnostic ultrasound system (EPIQ 7, Phillips Ultrasound, Inc., Washington, USA). All measurements were completed by the same investigator in conformance with current guidelines.^13^ At rest, during the initial (25 W load), maximum achieved load, and the recovery phase, conventional echocardiographic parameters, the volume and function of LA were assessed, together with the evaluation of the severity of MR, tricuspid regurgitation (TR), and SPAP.

The severity of MR was identified according to quantitative parameters – effective regurgitant orifice area (EROA) and regurgitant volume (RVol) - which were obtained according to existing recommendations.^14,15^ MR was assessed as moderate to severe with EROA 0.30–0.39 cm^2^ and RVol 45–59 mL.^15^ SPAP was calculated using the Bernoulli equation (SPAP = 4 × (TR V_max_)^2^ + 5 mmHg assigned to the right atrium (RA) pressure).^16^ Pressure of RA was determined according to the collapsing of the inferior vena cava during respiratory phases.^13^

LA volumes were measured using the area–length method in the apical four and two chambers’ views. In different phases of the cardiac cycle, the following volumes were measured: (1) maximal volume of LA (LA Vol_max_): just before the opening of MV at the end of the LV systole (ECG – at the end of the T wave); (2) minimal volume of LA (LA Vol_min_) – immediately after closure of MV, at the end of LV diastole; (3) LA volume just before atrial contraction (LA Vol_p_) – just before *p* wave in ECG. All measurements were indexed by the body surface area (BSA), which was calculated using the Du Bois formula. From these volumes, the dynamic volumes and fractions, related to different phases of the LA function, were calculated:^17^ *Reservoir function of LA*: LA total emptying volume = Vol_max_ – Vol_min_; LA total emptying (reservoir) fraction = (Vol_max_- Vol_min_)/Vol_max_ x 100 %; *Conduit function of LA:* LA passive emptying volume = Vol_max_ – Vol_p_; LA passive emptying (conduit) fraction = (Vol_max_ – Vol_p_)/Vol_max_ x 100 %; Conduit volume = LV stroke volume – (LA Vol_max_ – Vol_min_); *Contractile function of LA*: LA active emptying volume = Vol_p_ – Vol_min_; LA active emptying (contractile) fraction = (Vol_p_ – Vol_min_)/Vol_p_ x 100 %. The LA EF was calculated as the LA EF = LA active emptying volume/Vol_p_.^17^

### Stress echocardiography

Exercise stress echocardiography was performed using a bicycle ergometer (Ergoline GmbH, Germany) according to the existing recommendations^3^ in a semi-supine position starting with a 25 W workload and gradually increasing workload by 25 W every 3 min.^18^

### Speckle–tracking echocardiography

Myocardial deformation parameters were obtained by an off–line speckle–tracking analysis (using Philips QLAB 13.0 program). LV longitudinal strain (GLS) was evaluated from 4, 3 and 2 chambers apical views. Using an automatic “autoLA strain” analysis function, the deformation parameters of LA were obtained in four-chamber apical views.^19,20^ The global longitudinal strain of LA was divided into 3 phases: LA strain during the reservoir phase (LAS-r), positive value; LA strain during the conduit phase (LAS-cd), negative value; and LA strain during contractile phase (LAS-ct), negative value. The stiffness index of LA was calculated as E/e′ divided by LAS-r.^21^ The ratio between early diastolic filling (E wave) and LAS-r was considered the LA filling index.^22^ Extrasystolic cardiac cycles and segments with limited quality and/or poor tracking were excluded from the myocardial strain analysis.

### Laboratory testing

In 35 patients of the MR group NT-proBNP test was performed by collecting venous blood samples a few minutes before the stress test. Blood samples were first centrifuged in the laboratory of the Laboratory Medicine Clinic of Kaunas University of Health Sciences Hospital, then stored in a freezer at −80°C. After collection, all samples were analysed using the “PATHFAST” analyser (Mitsubishi Chemical Mediene Corporation, Tokyo, Japan) using immunological chemiluminescence.

### Statistical analysis

Statistical analysis was performed using IBM SPSS 27.0 software. Data distribution of the continuous variables was determined by the Shapir–Wilk test. Quantitative data were presented as mean and standard deviation (SD) if normally distributed, and if the distribution was abnormal or the sample size was small (<30) the median and interquartile area (first quartile – third quartile) were used. Qualitative data was presented by frequencies and frequencies in percentage (*N* (%)). Characteristics of the two groups were compared using Mann–Whitney U test or Student‘ t-test. The correlation between two related variables was calculated using Pearson‘s or Spearman‘s correlation coefficients (r). If *p* < .05, data was considered statistically significant. To prognose the occurrence of EIPH, receiver – operating characteristic (ROC) analysis was performed.

## Results

91 asymptomatic subjects (64 (70.33 %) females and 27 (30.7 %) males; mean age 60.92 (10.89) years) with preserved LV EF were enrolled in this study. There were no differences in demographic, anthropometric indicators and clinical data between MR and control groups (Table 1). NT–proBNP concentration was 128 [75–266.50] pg/mL in patients with MR.

**Table 1.** Demographic, anthropometric and clinical parameters in MR and control groups.

	MR group (*n* = 60)	Control group (*n* = 31)	*p* value
Age, years (SD)	61.45(11.99)	59.9(8.66)	0.526
Sex, male, *n* (%)	18(30.5)	9(31.0)	0.960
Body surface area, m^2^ (SD)	1.85(0.21)	1.93(0.18)	0.069
Heart rate at rest, beats/min (SD)	72.37(10.84)	72.68(10.69)	0.987
Systolic arterial blood pressure at rest, mmHg (SD)	137.45(19.78)	137.16(20.36)	0.844
Diastolic arterial blood pressure at rest, mmHg (SD)	82.0(2.10)	82.19(10.78)	0.826
History of atrial fibrillation, *n* (%)	16(26.67)	3(9.68)	0.059
Arterial hypertension, *n* (%)	45(75.0)	22(70.97)	0.679
Diabetes mellitus, *n* (%)	1(1.67)	3(9.68)	0.077
Dyslipidaemia, *n* (%)	33(55.0)	21(67.74)	0.241
ACEi usage, *n* (%)	26(43.33)	15(48.39)	0.646
ARB usage, *n* (%)	7(11.67)	4(12.90)	0.864
BAB usage, *n* (%)	45(75.0)	20(64.52)	0.294
CaCB usage, *n* (%)	6(10.0)	6(19.4)	0.211
Diuretics usage, *n* (%)	22(36.67)	10(32.26)	0.676

MR: mitral regurgitation, ACEi: angiotensin converting enzyme inhibitor; ARB: angiotensin receptor blocker; BAB: beta adrenoreceptor blocker; CaCB: calcium chanel blocker.

Values are expressed as number (%), mean (standart deviation).

LV GLS was lower in patients with MR, during all stages of stress, however, the difference was not significant (*p* > 0,5). LV GLS during peak stress correlated with NT–proBNP concentration (r = −0.446, *p* = .022).

**Figure 1.**
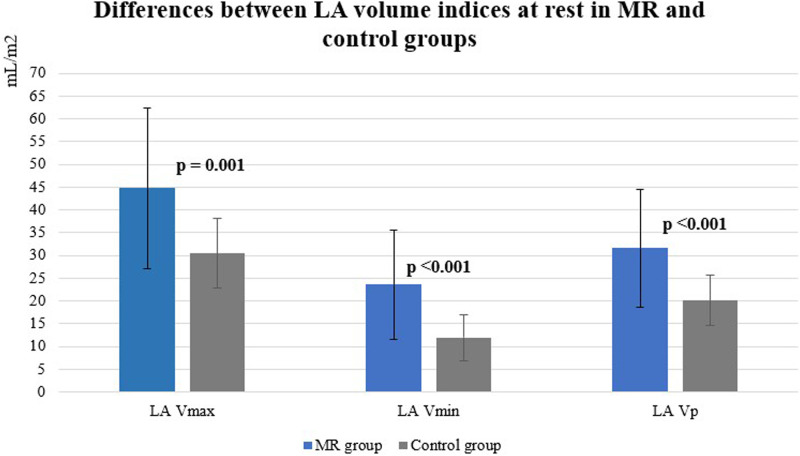
Differences between LA volumes at rest in MR and control groups. MR: mitral regurgitation, LA: left atrium, V_max_: maximal volume, V_min_: minimal volume, V_p_: volume just before atrial contraction.

At rest all LA volume indices were significantly higher in the MR group (Figure 1).

At rest LA functional parameters were lower in patients with MR than in controls (reservoir fraction 48.33 % (10.59) versus 61.35 % (8.67), *p* < .001, respectively; conduit fraction 29.57 % (8.47) versus 35.22 % (9.25), *p* = .014, respectively; contractile fraction 26.38 % (13.26) versus 41.58 % (12.13), *p* < .001, respectively) and LA filling index was higher (2.86 (1,29) versus 2.22 (0,74), *p* = .030). However, LA deformation parameters were similar in both groups at rest (*p* > .05).

Parameters of LA at rest were related to SPAP in different phases of stress: LA reservoir and conduit volume indices at rest correlated with resting SPAP (r = 0.329, *p* = .028 and r = 0.357, *p* = .016, respectively), while resting LA reservoir and contractile fractions correlated with SPAP during peak stress (r = −0.442, *p* = .002 and r = −0.361, *p* = .012, respectively).

**Figure 2.**
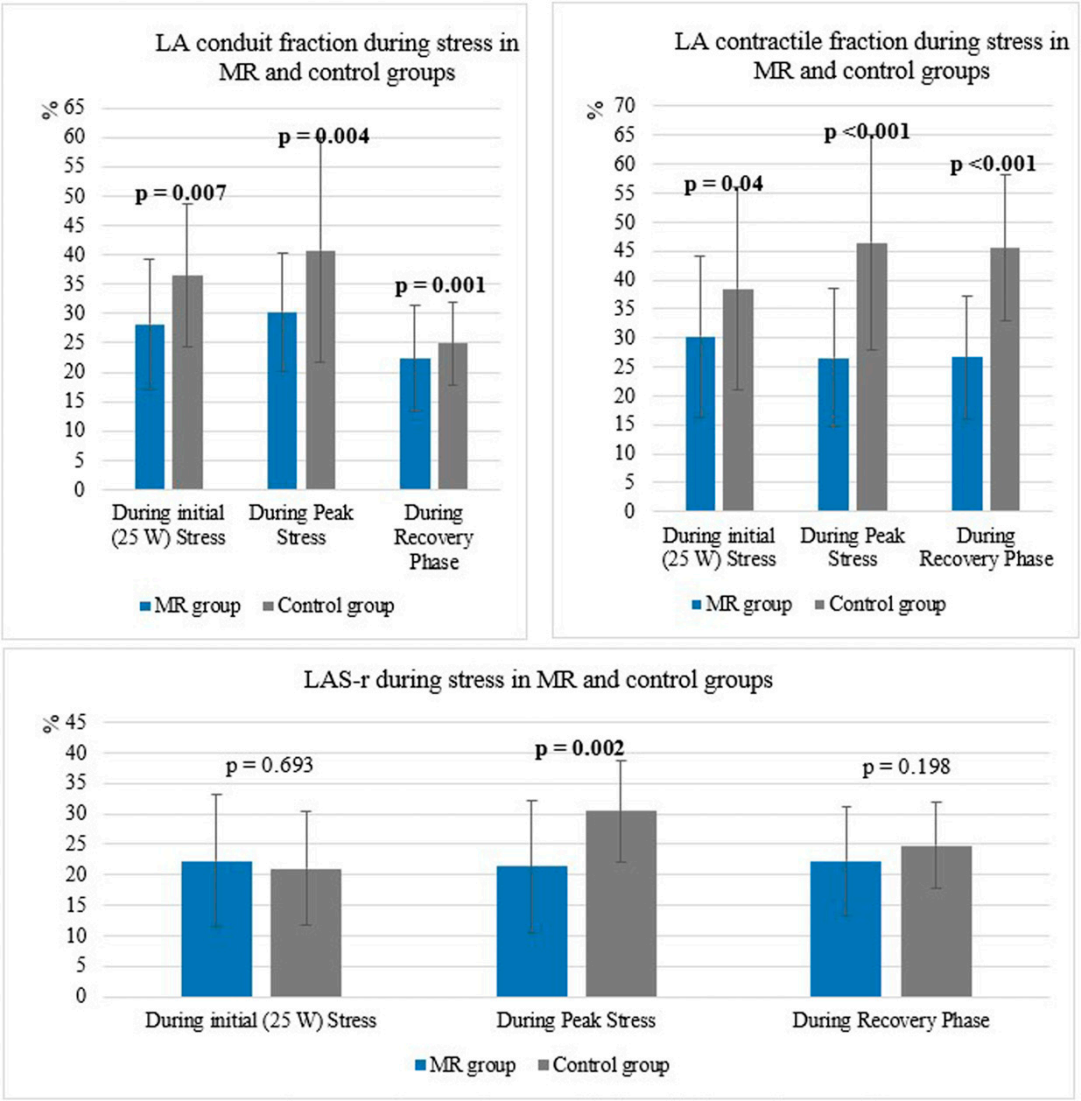
LA parameters in MR and control groups during different phases of the stress. MR: mitral regurgitation, LA: left atrium, LAS-r: strain during the reservoir phase.

During peak stress LA conduit, contractile fractions and reservoir strain were lower in patients with MR than in controls (Figure 2).

The correlations between SPAP at peak stress and LA filling index during initial stress (r = 0.348, *p* = .021) and during peak stress (r = 0.304, *p* = .038) were obtained.

The correlations between LV GLS at rest and indexed LA Vol_max_ (r = 0.287, *p* = .019), LA Vol_min_ (r = 0.331, *p* = .007), LA Vol_p_ (r = 0.325, *p* = .008) and LA reservoir fraction (r = 0.285, *p* = .020) during the phase of maximal achieved stress were obtained.

Higher LA volume indices during all stages of stress, lower LA contractile and reservoir functions during peak stress were related to higher NT–proBNP concentration (Table 2).

**Table 2.** Correlation of LA parameters with NT-proBNP.

LA parameter	At rest	During initial (25 W) stress	During peak stress	During recovery
LA Vol_max_	**r = 0.441**	**r = 0.518**	**r = 0.476**	**r = 0.421**
	**p = .019**	**p = .007**	**p = .010**	**p = .029**
LA Vol_min_	**r = 0.527**	**r = 0.630**	**r = 0.536**	**r = 0.511**
	**p = .004**	**p = .001**	**p = .003**	**p = .006**
LA Vol_p_	**r = 0.406**	**r = 0.694**	**r = 0.454**	**r = 0.494**
	**p = .032**	**p < .001**	**p = .015**	**p = .009**
LAreservoir fraction	r = −0.118	**r = –0.403**	**r = –0.444**	r = −0.374
	*p* = .359	**p = .041**	**p = .018**	*p* = .055
LA conduit fraction	r = −0.198	r = −0.127	r = −0.033	r = −0.174
	*p* = .313	*p* = .307	*p* = .866	*p* = .386
LA contractile fraction	r = −0.357	r = −0.327	**r = –0.436**	r = −0.327
	*p* = .062	*p* = .103	**p = .020**	*p* = .096

Bold values are statistically significant. LA: left atrium; Vol_max_: maximal volume; Vol_min_: minimal volume; Vol_p_: volume just before atrial contraction.

One-third (*n* = 20 (33.33 %)) of the MR group developed EIPH. Their age, gender, HR, or resting systolic and diastolic BP did not differ from patients without EIPH (*p* > .05). There were higher LA Vol_max_ (48.81 [36.85–65.29] mL/m^2^ vs 36.59 [30.75–45.62] mL/m^2^, *p* = .013 at rest and 57.84 [45.23–68.70] mL/m^2^ vs 45.79 [38.82–52.47] mL/m^2^, *p* = .017 during peak stress), Vol_min_ (24.97 [20.53–44.23] mL/m^2^ vs 17.66 [14.22–23.49] mL/m^2^, *p* = .002 at rest and 33.53 [22.00–45.55] mL/m^2^ vs 20.86 [16.22–26.05] mL/m^2^, *p* = .003 during peak stress), Vol_p_ (34.16 [24.83–49.23] mL/m^2^ vs 26.11 [21.64–32.09] mL/m^2^, *p* = .026 at rest and 42.49 [31.17–53.06] mL/m^2^ vs 31.14 [24.41–37.26] mL/m^2^, *p* = .013 during peak stress) in PH subgroup. Furthermore, patients with EIPH had lower LAS-r during peak stress (15.8 [10.20–22.80] % vs 25.4 [16.3–29.55] %; *p* = .008).

According to ROC analysis, the LA volume indices, LA EF, and filling index at rest could be informative predictors of EIPH (Table 3).

**Table 3.** ROC analysis of resting LA parameters for predicting EIPH in MR group.

Parameters at rest	Cut-off value	AUC [95 % CI]	Sensitivity,%	Specificity, %	EIPH-/+	*p* value	Or [95% PI]
LA Vol_max_, mL/m^2^	>40.59	0.712[0.562–0.963	72.2	60.6	13 (39.39 %)/13 (72.22 %)	**0.025**	4.00[1.151–13.89]
LA Vol_min_, mL/m^2^	>19.1	0.759[0.624–0.898	83.3	63.6	13 (39.39 %)/15 (83.33 %)	**0.003**	7.692[1.854–31.911]
LA Vol_p_, mL/m^2^	>28.37	0.690[0.526–0.854]	72.2	66.7	11 (33.33 %)/13 (72.22 %)	**0.008**	5.200[1.475–18.332]
LA EF, %	<20.87	0.694[0.541–0.846]	72.2	72.7	9 (27.27 %)/11 (64.71 %)	**0.010**	4.889[1.393–17.159]
LA filling index	>0.29	0.712[0.569-0.855]	78.9	58.8	14 (41.18 %)/15 (78.95 %)	**0,008**	5.357[1.464–19.603]

Bold values are statistically significant. LA: left atrium; MR: mitral regurgitation; EIPH: exercise indused pulmonary hypertension; AUC: area under the curve; CI: confidence interval; OR: odds ratio; Vol_max_: maximal volume; Vol_min_: minimal volume; Vol_p_: volume just before atrial contraction; EF: ejection fraction.

## Discussion

In this study we assessed parameters of LA at rest and during exercise stress in patients with asymptomatic moderate-to-severe MR and preserved LV EF. Significant correlations between LA parameters and LV GLS, NT-proBNP concentration and SPAP were obtained. Moreover, LA parameters were found to be significant predictors of EIPH.

Consistent with Alexander N. Borg and co-authors,^17^ we observed higher LA volume indices across all cardiac cycle phases in the MR group at rest and during stress. Prior research has linked LA dilation to heightened LV pressure,^23^ more severe diastolic dysfunction,^24^ increased incidence of atrial fibrillation, and worse prognosis in the general population.^10,25,26^ Some authors have suggested that LA volume index ≥40 mL/m2 may serve as predictive factor for surgery indications.^8^ Furthermore, we noticed reduced LA conduit, reservoir, and contractile fractions in patients with primary MR. Initially, LA enlargement occurs in response to early-stage MR to enhance contractile capacity,^17^ but lately, chronic MR induces inflammatory changes and interstitial fibrosis,^27^ resulting in functional impairment and heart tissue no longer provoking a Frank-Starling response.^28^ Nonetheless, strains between various LA phases in the MR group did not diminish, suggesting still underdeveloped myocardial fibrosis. This is further supported by our finding that the calculated LA stiffness index did not significantly differ between MR and control groups during all phases of stress. Giulia Elena Mandoli et al. demonstrated the correlation of histologically confirmed LA fibrosis with LA longitudinal strain in primary MR patients, (245), and non-invasive indices of LA fibrosis have also shown a significant correlation with the LA stiffness index.^29^

We demonstrated correlations between NT-proBNP concentrations and LA volume indices for different phases of the cardiac cycle during all stages of stress. These results are consistent with the findings of prior research indicating an association between LA volume index and NT-proBNP concentrations, even in the presence of normal LV EF.^30,31^ This can be explained by the fact that larger LA volumes lead to more wall stretching and heightened synthesis of natriuretic peptides. This only proves the importance of LA volume indices in the assessment of early myocardial damage. Additionally, Thierry Le Tourneau et al. found that resting SPAP was most strongly associated with LA volume (*p* = .003), deceleration time (*p* < .0001), and E/e′ ratio (*p* < .0001),^32^ while Andrea Barbieri et al. demonstrated that resting SPAP was dependent on LA size (*p* < .0001).^33^ Similarly, we obtained a significant correlation of all cardiac cycle phase LA volumes (LA Vol_max_, Vol_min_, Vol_p_) and resting SPAP (*p* < .001). In chronic MR, the LA is overloaded with increasingly larger volumes, resulting in dilation. This LA overload can elevate pulmonary circulation pressure and lead to PH development. EIPH was not included among the indications for surgery recommended by the European Society of Cardiology, due to a lack of sufficient evidence linking it to worse postoperative outcomes,^14^ but our study revealed that one-third of primary MR patients developed EIPH, which can be accurately predicted by other indicators of poor prognosis such as increased resting LV volumes, stiffness indices, and LV EF. To identify pre-clinical indicators of myocardial damage that could signal the need for surgery, leading to better outcomes, further studies are needed to evaluate the long-term outcomes and prognostic value of these findings.

## Limitations

The main limitations of the study, which may have contributed to the inaccuracies of the study, were the small sample size and unequal groups, the assessment of PH by echocardiography rather than invasive right heart catheterisation. Finally, due to the limited duration of the study, our work did not assess the patients’ outcomes and the predictors of these outcomes on resting and exercise echocardiography.

## Conclusions

In patients with primary moderate to severe MR and preserved LV EF the most informative parameters of subclinical myocardial damage were higher LA volume indices, decreased LA EF and conduit fraction at rest and during stress. LA contractile and reservoir function during peak stress as well as LA volume indices during all phases of the stress had significant correlations with NT-proBNP. LA volume indices, EF, and filling index are related with EIPH in patients with primary MR.
